# Femoral and tibial cementless fixation neither increases blood loss nor impedes early functional recovery: A randomized controlled trial

**DOI:** 10.3389/fsurg.2022.1079120

**Published:** 2023-01-04

**Authors:** Jian Cao, Kai Liao, Ze-xi Li, Duan Wang, Jia-li Chen, Hao-yang Wang, Zong-ke Zhou

**Affiliations:** ^1^ Department of Orthopedics, Orthopedic Research Institute, West China Hospital, Sichuan University, Chengdu, China; ^2^ Department of Radiology, West China Hospital, West China Hospital, Sichuan University, Chengdu, China; ^3^ West China School of Medicine, Sichuan University, Chengdu, China; ^4^ West China School of Nursing, Sichuan University/Department of Orthopedics, West China Hospital, Sichuan University, Chengdu, China

**Keywords:** cementless fixation, cemented fixation, blood loss, drainage, early functional recovery, total knee arthroplasty

## Abstract

**Background:**

Whether cementless fixation on femoral and tibial components increases blood loss during total knee arthroplasty (TKA) is unclear. The purpose of this randomized controlled trial was to compare blood loss and early functional recovery between patients who underwent cementless or cemented TKA.

**Methods:**

Between November 2021 and April 2022, sixty-one eligible patients at our medical center were randomized to cementless and cemented group. The primary outcome was total blood loss (TBL). Secondary outcomes were drainage, knee swelling, anemia, transfusion, hematological indicators, early functional recovery, and postoperative complications. The early functional recovery included range of motion (ROM), Hospital for Special Surgery (HSS) score, walking distance.

**Results:**

A total of 61 patients were analyzed, of whom 30 underwent cementless fixation. On postoperative day 1, the mean TBL was 394.39 ml (SD 182.97 ml) in the cementless group and 382.41 ml (SD 208.67 ml) in the cemented group (*P* = 0.863). By postoperative day 3, the corresponding mean TBL was higher at 593.48 ml (SD 230.04 ml) and 603.80 ml (SD 213.16 ml) (*P* = 0.751). The two groups did not differ significantly in drainage, knee swelling, anemia, levels of hemoglobin or hematocrit or platelets, ROM, HSS score, walking distance, or rates of transfusion or postoperative complications.

**Conclusions:**

Cementless fixation on femoral and tibial components during TKA does not increase blood loss or impede early functional recovery, which suggests that clinicians need not worry about blood loss and early functional recovery when deciding what type of fixation to perform during TKA.

**Trial registration:**

Number: ChiCTR2100052857; Date: November 6, 2021.

## Introduction

Total knee arthroplasty (TKA) is a successful surgical procedure to relieve pain, correct deformities, and restore physical function. Aseptic loosening is the most common reason for failure in TKA and is a major concern (1). This has led some clinicians to consider cementless fixation instead of standard cement fixation in order to achieve long-lasting biological fixation of the implant to the bone (2). The available evidence suggests that cementless fixation is associated with lower risk of aseptic loosening than cemented fixation in TKA (3–5).

However, some authors have noted that the absence of cement during TKA fails to prevent bleeding from cancellous bone (2) or from veins and sinusoids (6). Thus, the potentially greater blood loss in cementless fixation is also a clinical consideration in the choice of fixation type. In fact, two randomized controlled trials (7, 8) indicated femoral or tibial cementless fixation did not influence blood loss. But to our knowledge, whether cementless fixation on femoral and tibial components increases total blood loss (TBL) during TKA remains inconclusive: four studies (9–13) concluded that it does, while two concluded that it does not (14). A recent meta-analysis (3) pooled higher TBL in femoral and tibial cementless fixation, but only included three randomized controlled trials with blood loss as the secondary outcome (9, 10, 14) and two were conducted more than 20 years ago. It is well known that great progress has been made in perioperative blood management based on the implementation of the “Enhanced Recovery After Surgery” program (15) within the last decade, and it is intriguing to re-evaluate the blood loss between the two fixation types.

Therefore, we conducted a prospective, randomized controlled trial to compare blood loss between patients who underwent cementless or cemented fixation on femoral and tibial components. In addition, we compared the two approaches in terms of early functional recovery.

## Materials and methods

This prospective, single-center, randomized controlled trial was approved by the Ethics Committee at our institution, and it was registered at the Chinese Clinical Trial Registry (ChiCTR2100052857). Written informed consent was obtained from participants before enrollment. Data in the trial were collected, analyzed and reported in conformity with the Consolidated Standards of Reporting Trials (CONSORT) Statement.

### Participants

We consecutively recruited patients 18–80 years old at our institution who were diagnosed with end-stage knee osteoarthritis and who underwent primary unilateral TKA between November 2021 and April 2022. Patients were excluded if they had a history of knee surgery, except arthroscopy; severe osteoporosis [The threshold of bone density value below the −2.5 SD of T-score, determined by dual-energy x-ray absorptiometry, and the presence of fragility fractures (16)] or bone defects; a history of deep venous thrombosis (DVT) or pulmonary embolism (PE); hematopoietic or hemorrhagic disorder; current use of anticoagulant therapies (warfarin or heparin); hemoglobin (Hb) < 100 g/L or C-reactive protein >10 mg/L (17); acupuncture or puncture of the target knee within three months before TKA; uncontrolled hypertension; allergy to the prosthesis; or refusal to participate.

### Interventions, randomization, and blinding

Eligible patients were allocated a unique number and randomized to groups that received cementless or cemented femoral and tibial components using a computer-generated randomization table by one independent investigator not involved in the data collection and analysis. This table, without patient identifiers such as the name, assigned each participant with its unique trial number to a corresponding intervention allocation code. Another two investigators who were blinded to group allocation collected data, while a fourth investigator, also blinded to group allocation, conducted statistical analysis of the data. Only patients were blinded to group allocation.

### Surgical procedure and perioperative management

One senior surgeon performed all procedures using a standard medial parapatellar approach while patients were under general anesthesia. Femoral resection was performed using an intramedullary alignment guide; tibial resection, using an extramedullary alignment guide. All patients received fixed bearings and posterior stabilized prostheses (Just Medical Devices, Tianjin, China). The aforementioned femoral and tibial prostheses were coated with titanium to increase friction and induce bone ingrowth. Meanwhile, three-dimensional printing technology was used to construct a three-zone trabecular structure (the pore size, porosity, and elastic modulus of trabecular bone in the inner, middle, and outer zones were different) on the back of the tibial plateau to avoid aseptic loosening caused by uneven stress distribution.

A drainage tube was used until 24 h after surgery. Prophylactic intravenous antibiotic was applied within the first 24 h postoperatively. A standardized venous thromboembolism prevention protocol was adopted, involving intermittent inflatable calf pump, ankle dorsal and plantar flexion, as well as enoxaparin, which was administered as 2,000 IU subcutaneously 6 h postoperatively and then 4,000 IU once daily until discharge. Rivaroxaban (10 mg) was administered orally 10 days after discharge. Trained rehabilitation nurses instructed the patients to perform active range of motion (ROM) exercises, lower-extremity strength training and ambulation according to standard procedures.

### Perioperative multimodal blood management

Blood was managed intraoperatively using hemostasis with electrocautery, a tourniquet with pressure set around 240 mmHg, sealing of the femoral medullary canal with autologous bone, minimization of surgical trauma (All operations were performed by the same experienced joint surgeon, which can shorten the operation time and avoid excessive soft tissue release and repetitive osteotomy.), and maintenance of systolic blood pressure around 100 mmHg. Tranexamic acid was given as five intravenous doses at 2 h before surgery (2 g), then at 3, 6, 12, and 24 h after surgery (1 g each). Postoperative drainage-clamping was conducted for 4 h.

### Outcome measures

The primary outcome was TBL. Secondary outcomes included drainage, knee swelling, anemia, transfusion, hematological indicators, early function recovery, and postoperative complications.

TBL on postoperative days 1 and 3 was calculated as described by Gross and Nadler et al. (18, 19). The detailed information of the calculated TBL is presented in ①–④.

① TBL (ml) = patient’s blood volume (PBV) × (HCT_pre_−HCT_post_)/HCT_ave_;
② PBV (ml) = 1,000 × [K_1 _× height (m) + K_2 _× weight (kg) + K_3_] (K_1_ = 0.3669, K_2 _= 0.03219, and K_3_ = 0.6041 for men; and K_1_ = 0.3561, K_2_ = 0.03308, and K_3_ = 0.1833 for women);
③ HCT_pre_ = the initial preoperative HCT level; HCT_post_ = the HCT on the morning of the postoperative day; HCT_ave _= the average of the HCT_pre_ and HCT_post_;
④ If either reinfusion or allogeneic transfusion was performed, the TBL is equal to the loss calculated from the change in HCT plus the volume transfused.

A graduated cylinder was used to measure drainage at 12 and 24 h postoperatively. Knee swelling was measured in terms of the circumference at the superior pole of the patella preoperatively and on postoperative days 2 and 3. The hematological indicators of Hb, hematocrit (HCT) and platelet count (PLT) were evaluated preoperatively and on postoperative days 1 and 3, week 6, and month 3.

Early functional recovery was assessed in terms of ROM, score on the Hospital for Special Surgery (HSS) scale (20), and walking distance. ROM was calculated as flexion minus flexion contracture with a oniometer while the patient was in a supine position; it was measured preoperatively and on postoperative day 3, week 6, and month 3. HSS score was determined preoperatively and on postoperative week 6 and month 3. Walking distance was recorded preoperatively and on postoperative month 3 according to HSS score.

Anemia, transfusion, and complications were documented during the 90 days after surgery. Diagnostic criteria for anemia were postoperative minimum Hb ≤ 12 g/dl in men and Hb ≤ 11 g/dl in non-pregnant women. Anemia was subclassified as mild if postoperative minimum Hb > 9 g/dl; moderate, 6–9 g/dl; severe, 3–6 g/dl; or extremely severe, ≤30 g/dl (21). Transfusion was performed when postoperative minimum Hb < 7 g/dl, or when postoperative minimum was 7–10 g/dl and the patient showed anemia symptoms such as dizziness or fatigue (22). The following complications were documented during the 90 days after surgery: DVT, PE, infection, readmission or mortality.

### Statistical analysis and sample size

Data were analyzed using SPSS 26.0 (IBM, Chicago, IL, USA). The Shapiro-Wilk test was used to test whether data were normally distributed. Continuous data were reported as mean and standard deviation (SD) if they showed a normal distribution, or as median and interquartile range (IQR) if they showed a skewed distribution. Categorical data were reported as numbers and percentages. Intergroup differences in continuous variables were assessed for significance using an independent-samples *t*-test if data were normally distributed, or the Mann–Whitney *U* test if data showed skew or unequal variance. Intergroup differences in categorical variables were assessed using the Pearson chi-squared test or Fisher's exact test. Differences associated with *P* < 0.05 were considered statistically significant.

The sample size was estimated based on a previously reported mean significant difference in blood loss of 143.8 ml (10) between cementless TKA and cemented TKA. To expand the sample size, our definition of 110 ml (SD ± 150 ml) as the smallest clinically meaningful reduction in blood loss between two groups. A power analysis implied that 29 participants per group were needed to achieve the power of 0.8 at 0.05 significance level.

## Results

### Patient characteristics

**Figure 1:**
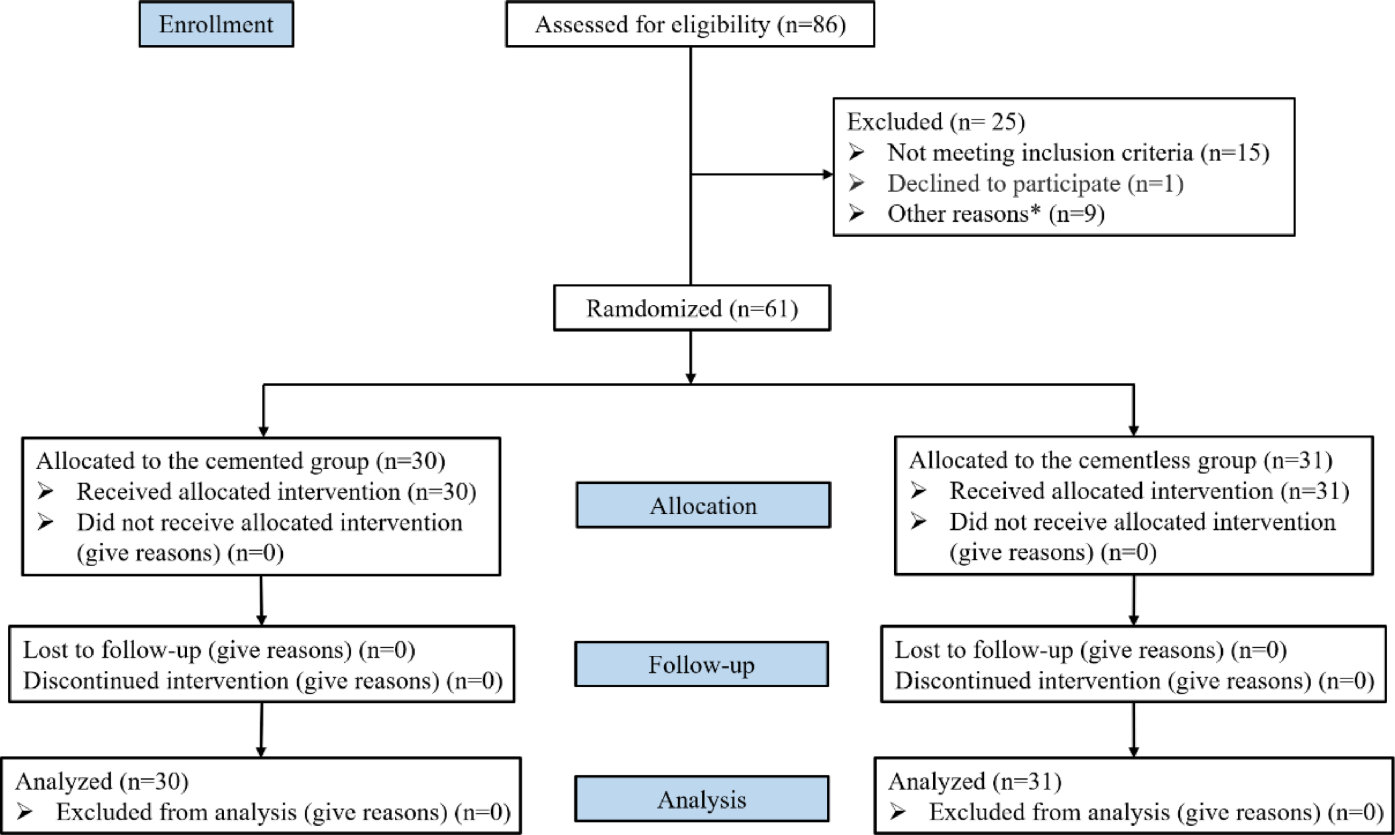
CONSORT (consolidated standards of reporting trials) flow diagram. Other reasons*: anticoagulant drugs (*n* = 3), severe osteoporosis or bone defect (*n* = 2), hemoglobin <100 g/L or CRP > 10 mg/L (*n* = 2), uncontrolled hypertension (*n* = 1), acupuncture or puncture 3 months before surgery (*n* = 1).

A total of 86 patients were assessed for eligibility, of whom 25 were excluded because they failed to meet the inclusion criteria or they fulfilled the exclusion criteria. The remaining 61 patients were randomly allocated to a cementless group (*n* = 30) or cemented group (*n* = 31). All patients were followed up for 3 months (Figure 1). The two groups did not differ significantly in baseline characteristics (Table 1).

**Table 1:** Baseline characteristics.

Characteristics	Cementless group (*n* = 30)	Cemented group (*n* = 31)	*P*-value*
Demographics			
Age^a^, y	64.7 (3.95)	65.03 (5.13)	0.778
Gender, No. (%)			0.687
Female	24 (80)	27 (87.1)	
Male	6 (20)	4 (12.9)	
Weight^a^, kg	63.29 (8.59)	64.03 ± 7.01	0.713
Height^a^, m	1.55 (0.06)	1.57 (0.06)	0.411
BMI^a^, kg/m^2^	26.28 (3.06)	26.08 (2.07)	0.765
PBV^a^, ml	2,906.03 (335.61)	2,908.42 (264.66)	0.975
Operation side, No. (%)			0.096
Right	12 (40)	19 (61.3)	
Left	18 (60)	12 (38.7)	
Operation time^a^, min	72.53 (16.15)	78.71 (14.41)	0.120
ASA clasification, No. (%)			0.704
I	0 (0)	0 (0)	
II	23 (76.7)	25 (80.6)	
III	7 (23.3)	6 (19.4)	
Hypertension, No. (%)	14 (46.7)	12 (38.7)	0.530
Type 2 DM, No. (%)	3 (10)	2 (6.5)	0.671
Preoperative clinical evaluation			
Knee circumference^a^, cm	37.58 (4.01)	37.38 (2.33)	0.817
Deformity, No. (%)			0.537
Varus	24 (80)	22 (71)	
Valgus	0 (0)	2 (6.5)	
Neutral	6 (20)	7 (22.6)	
ROM^b^, °	100 (78.75–110)	92.50 (85–110)	0.783
HSS score^a^			
Total	60.53 (13.8)	56.58 (10.95)	0.219
Pain	15.9 ± 5.83	14.19 ± 6.2	0.277
Function	14.6 (3.5)	12.71 (3.45)	0.060
Walking distance, No. (%)			0.079
Unlimited	0 (0)	0 (0)	
2.5–5 km	5 (16.7)	1 (3.2)	
0.5–2.5 km	14 (46.7)	11 (35.5)	
<0.5 km	11 (36.7)	19 (61.3)	
Unable	0 (0)	0 (0)	
Preoperative laboratory test^a^			
Hemoglobin, g/dl	13.5 (1.5)	13.5 (1.2)	0.947
Hematocrit, L/L	0.43 (0.04)	0.42 (0.03)	0.670
Platelet count, 10^9^/L	195.83 (61.28)	201.29 (52.1)	0.709
APTT, s	26.64 (2.78)	26.4 (2.3)	0.834
Prothrombin time, s	10.72 (0.81)	10.55 (0.55)	0.680

BMI, body mass index; PBV, patient's blood volume; ASA, american society of anesthesiologists; DM, diabetes mellitus; ROM, range of motion; HSS, hospital for special surgery; APTT, activated partial thromboplastin time.

^a^ Values are given as mean and standard deviation.

^b^ Values are given as median and interquartile range.

* *P* < 0.05 is significant.

### Blood loss

Mean TBL was 394.39 ml (SD 182.97 ml) in the cementless group and 382.41 ml (SD 208.67 ml) cemented group on postoperative day 1 (*P* = 0.863), which increased marginally to 593.48 ml (SD 230.04 ml) and 603.80 ml (SD 213.16 ml) on postoperative day 3 (*P* = 0.751).

**Figure 2:**
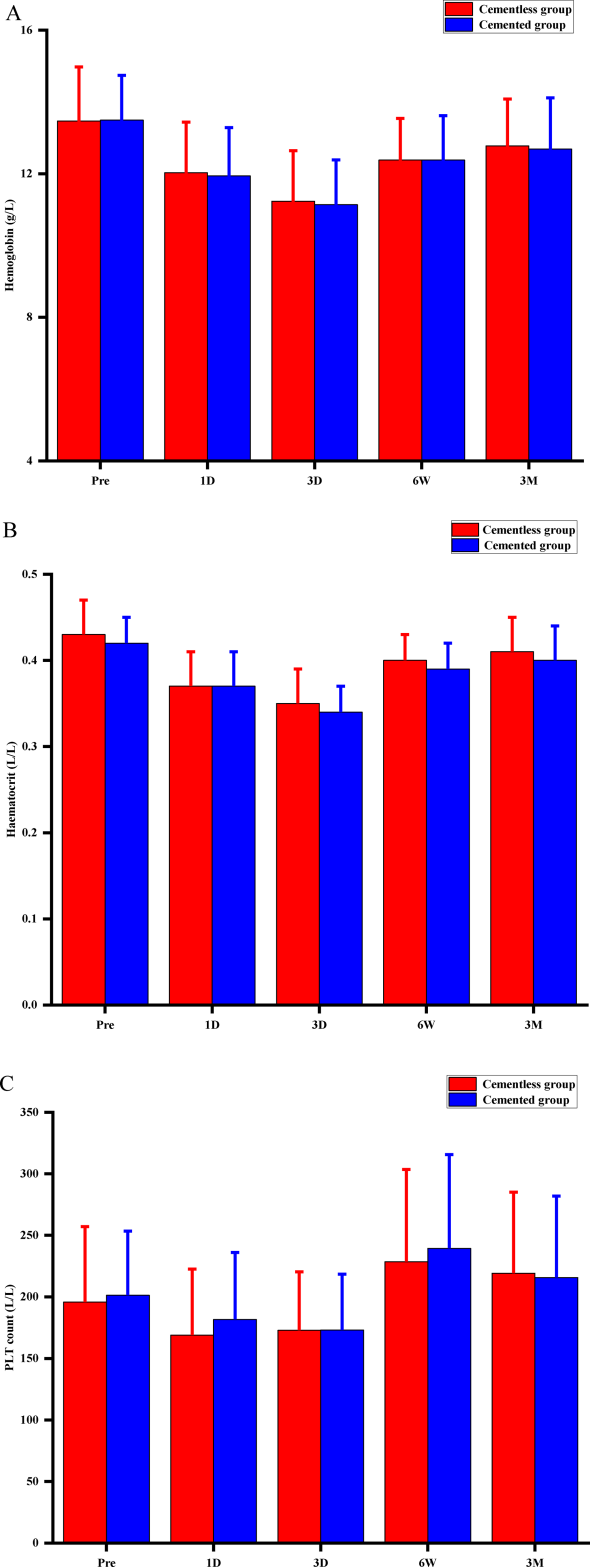
Perioperative hematological indicators for hemoglobin concentration **(A**), hematocrit (**B**), and platelet (PLT) count (**C**). Pre, preoperative; D, postoperative day; W, postoperative week; M, postoperative month; *P* > 0.05 at any time point.

The two groups did not differ significantly in knee circumference, drainage, or rate of anemia (Table 2), nor did they differ significantly in Hb, HCT, or PLT levels throughout the 90-day follow-up (Figure 2). The Hb and HCT levels of the final follow-up were significantly lower than preoperative values (*P* = 0.001, *P* = 0.003, respectively) between the two groups. No patients required intra- or postoperative transfusion.

**Table 2:** Blood Loss.

Characteristics	Cementless group (*n* = 30)	Cemented group (*n* = 31)	*P-*value*
Total blood loss^a^, ml			
Postoperative day 1	394.39 (182.97)	382.41 (208.67)	0.863
Postoperative day 3	593.48 (230.04)	603.80 (213.16)	0.751
Drainage^a^, ml			
Postoperative 12 h	91.67 (75.66)	87.1 (61.6)	0.960
Postoperative 24 h	123.33 (102.03)	115.48 (77.62)	0.983
Knee circumference^a^, cm			
Postoperative day 2	39.37 (4.16)	38.38 (2.63)	0.740
Postoperative day 3	40.6 (4.36)	40.54 (2.63)	0.801
Anemia, No. (%)			0.526
None	14 (46.7)	16 (51.6)	
Mild	15 (50)	12 (38.7)	
Moderate	1 (3.3)	3 (9.7)	
Transfusion, No. (%)	0 (0)	0 (0)	

^a^ Values are given as mean and standard deviation.

* *P* < 0.05 is significant.

### Early functional recovery and complications

The two groups did not differ significantly in ROM, total or functional HHS scores, or walking distance at any time point (Table 3). No patient in the study developed DVT, PE, or infection, nor were any cases of readmission or mortality recorded.

**Table 3:** Early Functional Recovery and Complications.

Characteristics	Cementless group (*n* = 30)	Cemented group (*n* = 31)	*P*-value*
ROM^a^, °			
Postoperative day 3	105 (100–110)	110 (100–110)	0.095
Postoperative week 6	112.5 (95–120)	107.5 (98.75–120)	0.445
Postoperative month 3	110 (100–120)	110 (95–120)	0.919
HSS total score^b^			
Postoperative week 6	79.23 (9.05)	77.26 (9.83)	0.418
Postoperative month 3	83.63 (9.19)	85.23 (6.04)	0.426
HSS function score^b^			
Postoperative week 6	14.7 (3.64)	14.61 (3.84)	0.928
Postoperative month 3	16.63 (3.46)	17.55 (2.83)	0.262
Walking distance, No. (%)			0.473
Unlimited	4 (13.3)	5 (16.1)	
2.5–5 km	10 (33.3)	12 (38.7)	
0.5–2.5 km	13 (43.3)	14 (45.2)	
<0.5 km	3 (10)	0 (0)	
Unable	0 (0)	0 (0)	
90-day complications, No. (%)			
Deep vein thrombosis	0 (0)	0 (0)	
Pulmonary embolism	0 (0)	0 (0)	
Infections	0 (0)	0 (0)	
Readmission	0 (0)	0 (0)	
Mortality	0 (0)	0 (0)	

ROM, range of motion; HSS, hospital for special surgery.

^a^ Values are given as median and interquartile range.

^b^ Values are given as mean and standard deviation.

* *P* < 0.05 is significant.

## Discussion

Our work diverges from the previous four studies involving femoral and tibial cementless or cemented fixation (9–12) in three important respects. One is that previous work reported significantly greater blood loss after cementless fixation than after cemented fixation, whereas we found similar blood loss between the two cases. Another is that previous work reported greater mean blood loss than our study, both for cementless fixation (700–1,300 vs. 394.4 ml) and for cemented fixation (200–600 vs. 382.4 ml). In addition, previous work reported high transfusion rates for both types of fixations, while no transfusion occurred in our study. From our point of view, these discrepancies may reflect advances in perioperative blood management, especially tranexamic acid is routinely used (23). A significant body of literature has demonstrated that tranexamic acid, as an antifibrinolytic agent, is an effective blood-sparing technique (24–33). The above four studies were conducted 20 years ago (9–12), implying that they used some relatively old blood management protocols, especially notably that all did not mention the application of TXA. In contrast, the current study used relatively proven perioperative multimodal blood management, including the use of tranexamic acid.

But it is worth noting that another recent study concluded that insufficient tranexamic acid may fail to reduce the additional intra-articular blood loss caused by femoral and tibial cementless fixation (13). In their study, a regimen of single-dose (1–2 g) topical or intravenous TXA was used in partial participants. They discovered that two fixations had similar TBL, but cementless fixation still resulted in more drainage during 24 h postoperatively. We relied on a multiple dosing of Intravenous tranexamic acid (the total dose is 6 g) in our study, reflecting findings from our medical center that multiple-dose intravenous tranexamic acid reduces blood loss in cemented TKA more effectively than single-dose tranexamic acid without increasing the risk of complications (29–31). We revealed similar TBL and postoperative 24-hour drainage, which proved their guess. Furthermore, no complications, readmission, or mortality occurred in our cohort up to 90 days after TKA.

In fact, Hood et al. (13) retrospectively found that there was no difference in change in Hb level from preoperatively to day 1 or day 2 postoperatively between the two fixation types on femoral and tibial components, which is consistent with a prospective study (14) (with a significant difference in baseline Hb level between the two groups). Our research reinforces their finding. Also, we observed no significant difference between the two groups in HCT and PLT levels. Importantly, we followed up with hematologic indicators four times over three months after surgery, and we found a consistent recovery in both groups, although Hb and HCT levels did not recover to preoperative values at the last follow-up.

Our findings support the results of two previous studies (7, 8) that reported no significant difference in blood loss, including Hb and HCT levels, volumes of postoperative suction drainage and TBL, and transfusion rate between cementless and cemented fixations. However, there was a main difference between their research and ours in the study design. In the study of Ishii et al. (8), the cementless group used cementless tibial components, and the cemented group used the cemented tibial components (tibial side). Meanwhile, both groups received cementless femoral components. Demey et al. (7) respectively applied the cementless and cemented femoral components to cementless and cemented groups (femoral side), and both groups received cemented tibial components. The similar findings of the three studies together reinforce the notion that cementless fixation on the tibial or/and femoral side does not significantly increase blood loss during TKA.

One further concern brought about by blood loss is that it may cause anemia and knee swelling, affecting respectively the patient's overall condition and local condition, which in turn affect early function recovery (31, 34–36). Our findings suggest that cementless TKA does neither increase risk of anemia or knee swelling nor compromise early functional recovery, which differs from a recent meta-analysis (3) that have suggested that a significantly worse postoperative Knee Society Score and ROM in cementless TKA. We believe that the consistent postoperative function recovery between the two groups in our study is closely related to similar blood loss. Unfortunately, previous studies that reported significantly greater blood loss with cementless TKA did not verify whether that translated to worse early functional recovery (9–14).

While this study is, to our knowledge, the first prospective trial to compare blood loss as primary outcome and early functional recovery between cementless and cemented TKA under modern perioperative blood management, its findings should be interpreted with caution given that we cannot identify with certainty why our results differ from those of several comparative studies published previously and the sample size of our study is relatively limited (The probability of making a type 2 error increases). In addition to advances in perioperative blood management, other explanations include advances in surgical techniques and prosthesis design. Future studies should explore the reasons why cementless fixation gives similar outcomes to cemented fixation, even though several studies indicate that it can lead to increased blood loss in some cases. Future work should also aim to optimize dose, timing and route of tranexamic acid administration. In any case, our findings should be verified and extended in larger, multi-site studies, preferably with even longer follow-up.

## Conclusions

Our study suggests that cementless fixation on femoral and tibial components during TKA neither increases blood loss nor impedes early functional recovery in comparison with cemented fixation, which implies that clinicians need not worry about blood loss and early functional recovery when deciding what type of fixation to perform during TKA.

## Data Availability

The original contributions presented in the study are included in the article/Supplementary Material, further inquiries can be directed to the corresponding author/s.
